# Design and Fabrication of a 2-Axis Electrothermal MEMS Micro-Scanner for Optical Coherence Tomography †

**DOI:** 10.3390/mi8050146

**Published:** 2017-05-05

**Authors:** Quentin A. A. Tanguy, Sylwester Bargiel, Huikai Xie, Nicolas Passilly, Magali Barthès, Olivier Gaiffe, Jaroslaw Rutkowski, Philippe Lutz, Christophe Gorecki

**Affiliations:** 1 FEMTO-ST Institute, CNRS UMR6174, University of Bourgogne Franche-Comté, 25000 Besançon, France; sylwester.bargiel@femto-st.fr (S.B.); nicolas.passilly@femto-st.fr (N.P.); magali.barthes@femto-st.fr (M.B.); olivier.gaiffe@femto-st.fr (O.G.); jaroslaw.rutkowski@hotmail.com (J.R.); philippe.lutz@femto-st.fr (P.L.); christophe.gorecki@femto-st.fr (C.G.); 2 Department of Electrical & Computer Engineering, University of Florida, Gainesville, FL 32611, USA; hkx@ufl.edu

**Keywords:** optical Micro Electro-Mechanical System (MEMS), Micro Optical Electro-Mechanical System (MOEMS), electrothermal actuation, torsion bar, dry photoresist, dual-reflective mirror, optical coherence tomography

## Abstract

This paper introduces an optical 2-axis Micro Electro-Mechanical System (MEMS) micromirror actuated by a pair of electrothermal actuators and a set of passive torsion bars. The actuated element is a dual-reflective circular mirror plate of 1 mm in diameter. This inner mirror plate is connected to a rigid frame via a pair of torsion bars in two diametrically opposite ends located on the rotation axis. A pair of electrothermal bimorphs generates a force onto the perpendicular free ends of the mirror plate in the same angular direction. An array of electrothermal bimorph cantilevers deflects the rigid frame around a working angle of 45${}^{\circ }$ for side-view scan. The performed scans reach large mechanical angles of 32${}^{\circ }$ for the frame and 22${}^{\circ }$ for the in-frame mirror. We denote three resonant main modes, pure flexion of the frame at 205 Hz, a pure torsion of the mirror plate at 1.286 kHz and coupled mode of combined flexion and torsion at 1.588 kHz. The micro device was fabricated through successive stacks of materials onto a silicon-on-insulator wafer and the patterned deposition on the back-side of the dual-reflective mirror is achieved through a dry film photoresist photolithography process.

## 1. Introduction

Optical Micro Electro-Mechanical System (MEMS) micro-scanners are exploited by a large variety of applications that usually require large displacement range, high operating frequencies, miniaturization, simplicity of packaging and integration. Various methods, such as piezoelectric, electrostatic, electromagnetic and electrothermal technologies [1] have been used to develop devices able to measure each application’s requirements. Among them, electrothermal actuation clearly stands out in terms of high performance, real time diagnosis, miniaturization of devices and endoscopy-based imaging. Although its working frequency is usually lower than for other actuation techniques, it still adequately reaches paces compatible with real time imaging [2]. MEMS electrothermal micro-scanners have a small size, high fill factor, high displacement range, low-voltage actuation and are relatively linear which makes them particularly adapted for in vivo endoscopic Optical Coherence Tomography (OCT) imaging applications [3].

**Figure 1:**
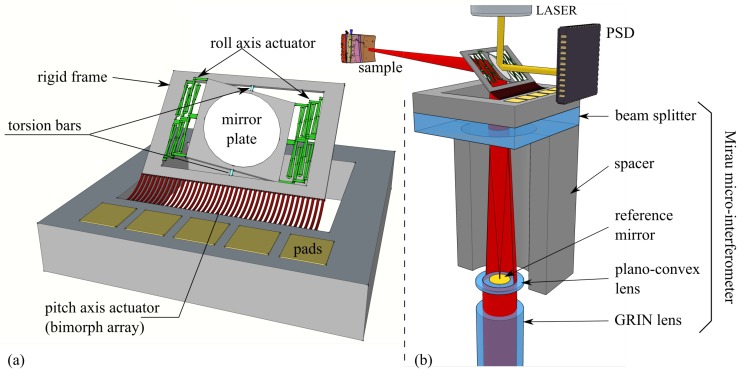
(**a**) Survey of the 2-axis Micro Electro-Mechanical System (MEMS) micro-scanning device. (**b**) Section plane of the different elements constituting the future endoscopic probe with the MEMS micro-scanner on top of the Mirau micro-interferometer for Optical Coherence Tomography (OCT) imaging process along with dynamical feedback control of the mirror position.

The micro-scanner proposed in this paper (shown in Figure 1a) was designed and fabricated in order to be, in a future perspective, embedded into a Swept-Source OCT (SS-OCT) endomicroscopic probe (Figure 1b) based on a Mirau micro-interferometer [4].

**Figure 2:**
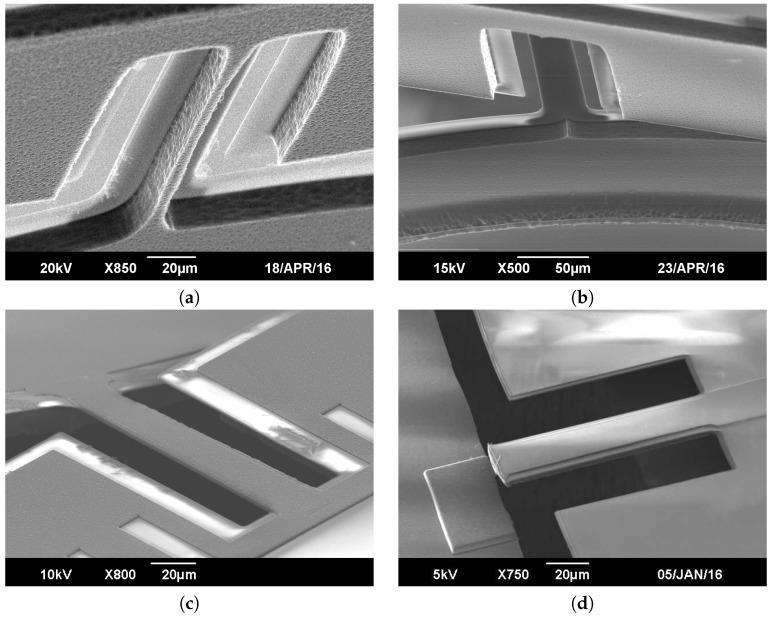
SEM pictures of torsion bar: (**a**) Sandwich bar from the backside before complete release. A narrow bridge of Si still holds the structure. (**b**) Sandwich bar from the backside after release. (**c**) Sandwich bar from the front side after release. (**d**) Example of Al-free torsion bar after release, broken under excessive torsion stress.

**Figure 3:**
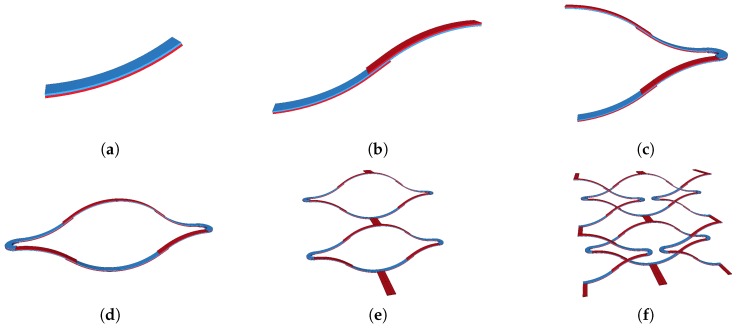
Schematic build up of the Meshed Inverted Series Connected (MISC) actuator. (**a**) Fundamental bimorph cantilever beam (tip-tilt and lateral displacement). (**b**) ISC actuator (Lateral shift). (**c**) Double S-shaped configuration (piston motion). (**d**) Pair of double S-shaped actuators in parallel (Stiffness and stability increased). (**e**) Cumbersome double actuator (increased displacement). (**f**) MISC actuator.

**Figure 4:**
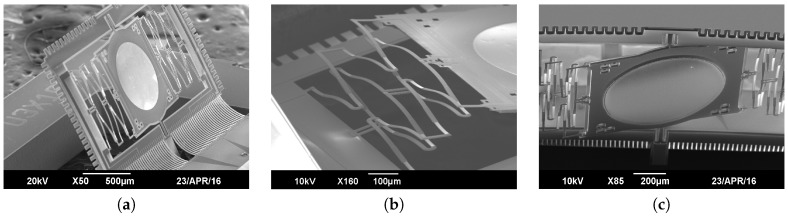
Scanning Electron Microscopy (SEM) pictures of the micro-scanner. (**a**) Overview of the front side of the mirror plate and the frame. (**b**) Detail of the MISC actuators after final release of the device. (**c**) Close-up view of the backward reflective side of the mirror plate.

Many MEMS micromirrors use a set of four electrothermal bimorph actuators located on the four sides of the central mirror plate [5,6,7]. During actuation or scanning, the center of these mirrors’ plate has to be partially maintained into a fixed position; first, by applying an offset voltage and second, by driving each pair of opposite actuators with a differential drive scheme [7]. However, the mirror plate is still subject to fluctuation with surrounding temperature and to uncontrolled changes due to vibrations or disturbances. In addition to these flaws, angular sensing mechanisms are usually unavailable, so that they are left uncontrolled [8] or with mere open-loop controls [9]. Concerning the few systems that demonstrate a close loop control, a single surface is used for both target operation and position sensing as in [10,11,12]. Conversely, for applications where one reflective side is to be exclusively dedicated to the main task as for OCT, phosphorescence or two-photon microscopy, exploiting the other side of the mirror is a reliable trade-off for direct position sensing compared to intermediate sensing methods [13,14], easy to be carried out at a macro scale in a preliminary stage. Our MEMS device is a 2-axis electrothermal scanning system characterized by a large scanning range, a torsion bar (Figure 2), a novel actuation mechanism (Figure 3 and Figure 4a) and a dual-reflective aluminum-coated mirror plate (Figure 4a,c). The Mirau micro-interferometer associated with the swept source performs an axial scan (A-scan). Once the micro-scanning device is embedded on top of it, two additional B-scan axes can be realized so that a 3D image can be obtained.

## 2. Design of the Device

This micromirror was designed to increase the stability of the in-frame mirror and to provide large scanning ranges over a large bandwidth at low driving voltage in order to allow in vivo operation and remedy to the lack of possibility of feedback control of the micromirrors. It shows off two reflective surfaces on both sides of the plate, appreciated for multi-use applications where the dynamics of the mirror plate need to be accurately controlled. Indeed, an optical position detector can sense the real-time angle on one of the two reflective sides. For actuation, a pair of meshed electrothermal actuators is associated to a set of torsion bars that helps keeping the central axis of the mirror steady. These structures are represented in Figure 1 in green and blue colors, respectively. The mirror plate is consequently tilted inside the frame using the pair of Meshed Inverted-Series-Connected (MISC) electrothermal actuators located on both sides of the plate. The actuators are inverted one from another and apply a force in the opposite direction on the mirror plate generating the rotation around the axis of roll. Meanwhile, the pair of torsional bars, that are collinear to the virtual axis of roll, maintains the axis of the mirror in the plane of the outer frame, thus bringing stability to the system over a wide frequency range. A Silicon On Insulator (SOI) substrate ensures mechanical and electrical bonding support to the outer frame which also bends out of plane. This rotative motion around an axis of pitch is made possible by a bimorph cantilever array (sketched in red color in Figure 1). Although it is actuated, the frame acts as a support for the in-frame mirror plate. The main frame and mirror plate are made of a 30 μm-thick SOI device layer.

### 2.1. Torsion Bar

The torsion bars are used to prevent the mirror plate from oscillating around the roll axis, thus restricting the motion to a pure rotation. The materials used for the torsion beams are limited to those used in the bimorph to simplify the fabrication process. They are made of a “sandwich” structure, composed of layers of SiO${}_{2}$/Pt/Al/SiO${}_{2}$ respectively. The torsion bars were purposely dimensioned so that the expression of the bending mode of the torsion rods is minimized and does not impact the torsional motion. The stiffness of the bending mode is reported in [5,15,16] and is related to the resonance frequency through Equations (1)–(6):

(1) $$k_{b}=\sqrt{\frac{Ewt^{3}}{4L^{3}}},$$

where *L*, *w*, *t* refer respectively to the length, width, and thickness of the torsion bar and *m* to the mass of the mirror plate. The frequency of the bending mode is given by:

(2) $$f_{b}=\frac{1}{2\pi }\sqrt{\frac{k_{b}}{m}}$$

The torsion mode stiffness of the system can be estimated from:

(3) $$k_{\phi }=2k_{t}+2\frac{L_{m}^{2}}{4}k_{b}$$

(4) $$\mathrm{with}\;k_{t}=\frac{\mu wt^{3}}{3L}.\left(1-\frac{192}{\pi^{5}}\left(\frac{t}{w}\right)\sum_{n=1,3,5,\ldots }^{\infty }\frac{1}{n^{5}}\tanh\left(\frac{n\pi t}{2w}\right)\right)$$

the free torsion stiffness as reported in [17,18]. Finally, the frequency of the torsion mode is given by [1]:

(5) $$f_{t}=\frac{1}{2\pi }\sqrt{\frac{k_{t}}{J_{t}}},$$

with $k_{t}=\frac{2\mu I_{t}}{L}$ where $I_{t}$ is the second moment of area of the torsion shaft, $J_{t}$ the moment of inertia of the mirror plate, $\mu =\frac{E}{2(1+\nu )}$ the shear modulus of elasticity, *E* the average Young’s modulus and ν the Poisson’s ratio. The torsion frequency can also be calculated via the second moment of area for a rectangular-sectioned bar given by [18]:

(6) $$I_{t}=wt^{3}\left(\frac{1}{3}-0.21\frac{t}{w}\left(1-\frac{t^{4}}{12w^{4}}\right)\right),$$

where *w* and *t* are respectively the width and thickness of the torsion bar.

**Figure 5:**
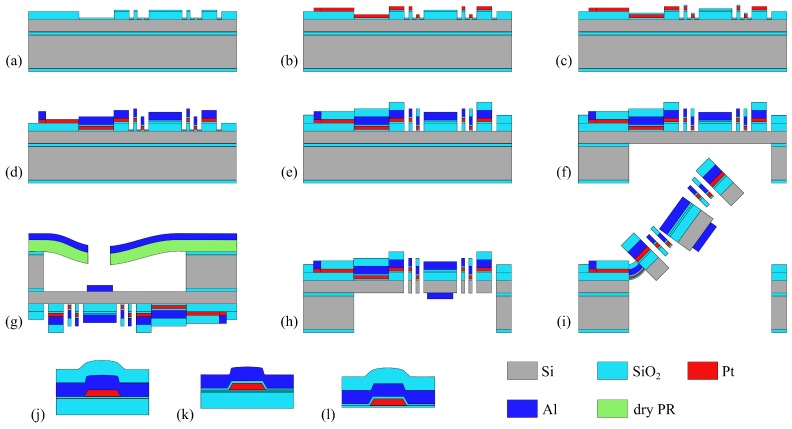
Fabrication steps (**a**) PECVD first layer of SiO${}_{2}$. (**b**) Sputter of the Pt heater. (**c**) PECVD of an insulation layer of SiO${}_{2}$ followed by via opening. (**d**) Evaporation of Al. (**e**) PECVD second layer of SiO${}_{2}$. (**f**) Anisotropic dry etch of the handle layer & BOX dry etch. (**g**) Lamination of dry PR & evaporation of Al. (**h**) Anisotropic etching of device layer from the front side. (**i**) Si isotropic etching to release. (**j**) Cross section of torsion bar. (**k**) Cross section of inverted bimorph. (**l**) Cross section of non-inverted bimorph.

The bending mode frequency of the torsion bar is chosen to be twice as high as its torsion mode frequency. To do so, the bar is 3.3 μm thick, 180 μm long and 28 μm wide. Figure 2 shows the torsion bar before and after release for different conditions and a schematic cross section of the torsion bar can be found in Figure 5j. The Si layer from the device layer located underneath the torsion bar (Figure 2a) does not remain in the released structure. Otherwise, it would hold the whole structure and eventually culminate in the breaking of the MISC electrothermal actuators. The layer of Al is sandwiched between the main layers of SiO${}_{2}$ and brings ductility to the torsion. The aluminum somewhat pushes away the yield stress breaking point of the structure making it more reliable regarding dynamical torsion and fatigue resistance. If not, the high residual stress initially induced in the actuators during fabrication would lead to fatal damages as pointed out in Figure 2d.

### 2.2. Electrothermal Actuation & MISC Actuators

The actuators are often cumbersome and are responsible for a much larger footprint of the final device than the size of the mirror plate. This issue has been tackled in some cases by modifying the shape of the actuators as in [19]. We present here an actuator based on Inverted-Series-Connected (ISC) electrothermal actuators as demonstrated in [7] but providing more flexibility and a higher displacement. It is a mesh of ISC actuators in series and in parallel that optimizes the space around the mirror plate to increase the displacement and the force of the actuators without degrading the fill factor. The principle of the meshed ISC (MISC) actuator is shown in Figure 3.

The MISC actuator is the latest evolution of four generations of shapes of electrothermal actuators: the single bimorph cantilever is the core element shown in Figure 3a and reported in [20,21]. In [6,7], bimorphs were connected in series as in Figure 3b,c including inverted and non-inverted bimorphs (whose cross sections are shown respectively in Figure 5k,l) to get rid of the tip-tilt effect, bypass the lateral shift and end up into a pure vertical translative motion called piston motion. These latter structures were then interconnected in parallel as in Figure 3d to increase the overall motion stability. Figure 3e shows an intermediate structure and was reported by [22]. The MISC actuator shown in Figure 3f is the structure actuating the micromirror and can be seen, as fabricated in Figure 4b. The torsion bars generate a counter momentum in the opposite direction of the momentum created by the two actuators. Hence, the actuators need to be able to provide a higher force and a larger displacement than that which can be provided by conventional ISC actuators. For a comparable space occupied, the MISC actuators provide a higher force, a larger displacement and a higher flexibility. This latter advantage is also highly appreciated during the release process and brings more suppleness for industrial fabrication where the dispersion of parameters on a single wafer can be significant. The bimorph is a sandwich of 1.1 μm of Al and 1 μm of SiO${}_{2}$. A thin heater layer of 1500 Å of Pt insulated in a sheath of thin SiO${}_{2}$ is wrapped between the Al and the SiO${}_{2}$ as shown in Figure 5c.

### 2.3. Dual-Reflective Mirror Plate

The mirror is coated with aluminum on both sides of the plate using E-beam evaporation. The deposition on the upper side is 1.1 μm thick and is performed during the same Al metalization as for the bimorphs. The Al layers of the front side mirror and of the electrothermal bimorph cantilevers are realized in one step using the same photomask to simplify the complete fabrication process. Therefore, the Al of the mirror plate has the same thickness as the layer of the bimorphs. The backside of the mirror is the side used to scan the focused laser beam and its smoothness is critical for the OCT image quality. Hence, the deposition is done at very low deposition rate (1.2 $Å\;s^{-1}$) while the substrate is being rotated at a speed of 10 rpm. SEM pictures of the reflective front and back side are shown respectively in Figure 4a,c.

## 3. Fabrication

The complete fabrication process is described in Figure 5. The devices are fabricated on an SOI wafer of 500 μm of handle layer, 30 μm of device layer and 1 μm of BOX. After a thorough clean up of the wafer, the first step (Figure 5a) consists of a deposition of 1 μm of Plasma-Enhanced Chemical Vapor Deposition (PECVD) SiO${}_{2}$ on the device layer which is subsequently wet etched to form the bottom layer of the non-inverted bimorphs, the hard frame, the torsion bars and the thermal bridges. It is then followed by another PECVD deposition of a thin layer of SiO${}_{2}$ as an insulator and a lift-off of platinum (Figure 5b) to pattern the heater throughout the actuators, the electrical paths and the pads.

The platinum is also used in the sandwich of the torsion bars. The three central pads control the inner actuators of the roll axis and are connected to the Al path of the bimorph array and isolated from its Pt layer via the SiO${}_{2}$ insulation film (Figure 5c). By doing so, the heat transfer generated by the current driven through the bimorph array is minimized. As shown in Figure 5d, a thick layer of 1.1 μm of Al is deposited by evaporation (to facilitate the lift-off process) following a photolithography of 3.5 μm of AZ nLOF2035 for the bimorphs, the mirror plate on the front side, the torsion bars, the electrical paths and the pads. We used pure Al, which was then protected by a thin coat of Cr of 150 Å to prevent oxidation.

A second layer of 1 μm of SiO${}_{2}$ is deposited by PECVD and patterned through RIE/ICP dry etch to form the top layer of the inverted bimorphs. This step is represented in Figure 5e.

The handle layer is anisotropically etched through DRIE to form the device’s backside cavity (Figure 5f). The exposed BOX is also etched with RIE/ICP until the buried face of the device layer is reached. Then Al is deposited onto the mirror plate’s backside by evaporation. A dry film photoresist DuPont ™ WBR2050 was laminated at 85 ${}^{\circ }C$ on the backside of the SOI wafer held by a carrier wafer before exposition.

The final release stage of the device divides into two substages respectively shown in Figure 5h,i. The first one consists of an anisotropic dry etch all the way through the device layer followed by an isotropic etch to release the actuators. The isotropic etch should not be performed longer than necessary to avoid ablating SiO${}_{2}$ from the deformable elements which could eventually damage or break them. The isotropic process time is interrupted when the frame and the inner actuators pop out of the plane. At that step, a plasma O${}_{2}$ can be used to get rid of the impurities remaining on the chip. An SEM picture of the micro scanner after release can be found in Figure 4a.

**Figure 6:**
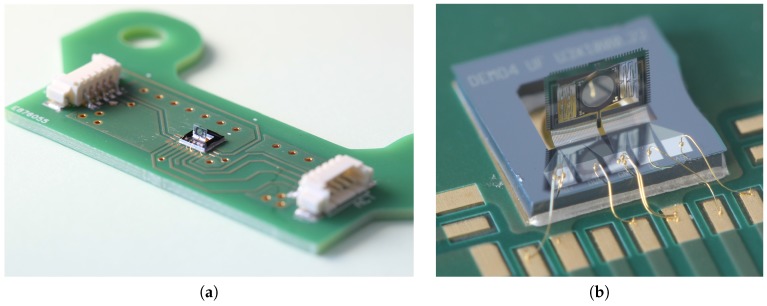
PCB support for the MEMS micro-scanner handling and testing. (**a**) Overview of the multi-use PCB with the micro device in the center, and connectors on both sides. (**b**) Zoomed-in picture of the micro device bonded onto the central gold pad with silver epoxy glue and wire bonding on Cu/Au pads for electrical routing.

Finally, several released chips are packaged onto a generic PCB support customized for handling and characterization of the micro-devices (Figure 6a). The micro-scanners are bonded onto the central Au pad with silver epoxy glue and electrically connected to the PCB pads by wire-bonding (Figure 6b).

## 4. Characterization

**Figure 7:**
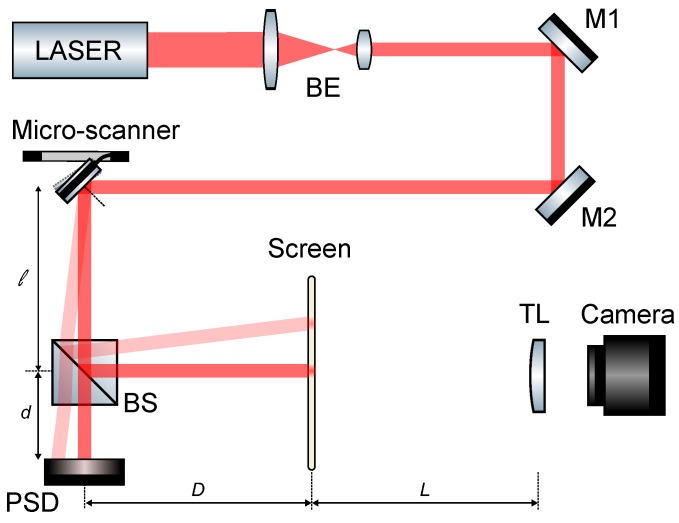
Optical setup implemented for the MEMS micro-scanner statical and dynamical characterization. BE: beam expander, M1, M2: mirrors, BS: beam splitter, PSD: Photo Sensing Detector, TL: tube lens.

After release, the electrical resistances of the roll axis actuators in parallel and the pitch axis actuator are 1.07 kΩ and 1.34 kΩ, respectively. The optical setup is shown in Figure 7.

**Figure 8:**
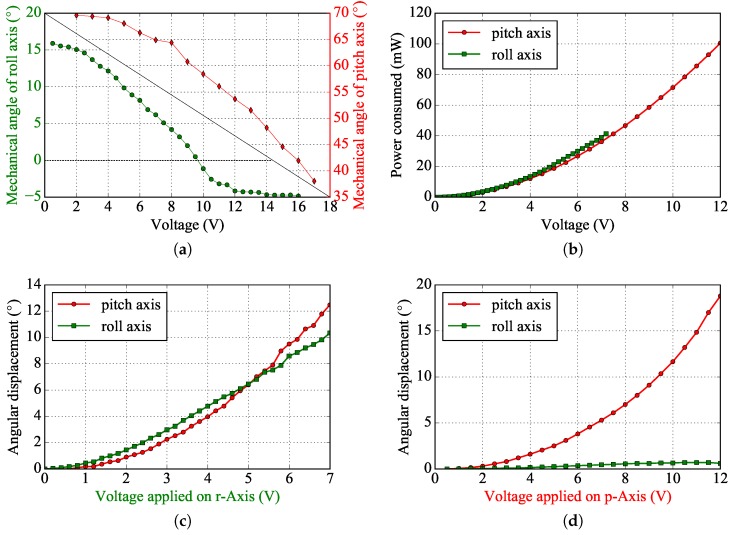
(**a**) Statical angular displacement of the mirror on the roll axis (in green) and of the frame on the pitch axis (in red). (**b**) Power consumption vs. voltage applied for both axes. (**c**) Statical relative angular displacement of both axes when only the roll axis is driven. (**d**) Statical relative angular displacement of both axes when only the pitch axis is driven.

A laser beam is directed onto the MEMS micromirror which reflects it towards a diffusing screen. The latter is observed from its backside by an ultra fast Phantom ™Miro M120 camera. The frame declines by 32${}^{\circ }$ from an initial angle of 70${}^{\circ }$ to a final angle of 38${}^{\circ }$ reached at a voltage of 17 V (178 mW) while the mirror plate achieves a mechanical sweep range of 22${}^{\circ }$ deflecting from an initial angle of 18${}^{\circ }$ to −4${}^{\circ }$ for a voltage of 16.5 V (188 mW) (Figure 8a). The characteristics of power consumption and angular displacement as a function of the voltage applied are also shown in Figure 8.

**Figure 9:**
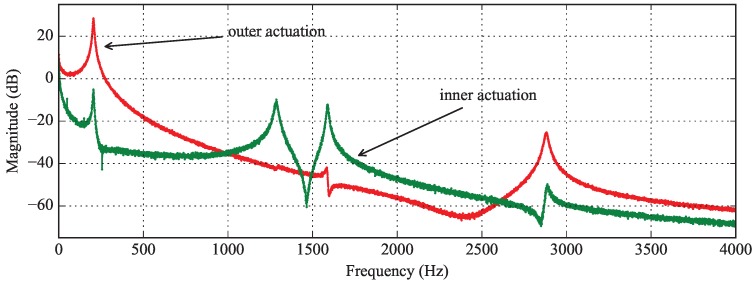
Superimposed frequency responses of the system when the voltage is applied on the pitch axis (outer actuator) in red and on the roll actuator (inner actuator) in green.

A Polytec ™MEMS Analyser was used to establish the frequency response of the micromirror. A white noise with an amplitude 1.5 V and an offset of 3 V was applied on the actuators one by one, and the magnitude of the deflection of the frame and the mirror plate was measured in dB. The Bode diagrams are shown in Figure 9. The coupling between the roll axis and the pitch axis is unilateral: when the inner actuator is driven, the heat is dissipated into the mirror plate, through the frame and through the bimorph array whose temperature increases at the same time, contributing into the cross-coupling of the two axes. In this situation, we observe four resonant modes: pure pitch motion at 205 Hz, pure roll motion of the torsional mirror plate at 1.286 kHz, a mode with both components at 1.588 kHz and a fourth mode that is less influential because of its high damping. Conversely, when the bimorph array is actuated, only the first pitch mode is observed at 205 Hz.

**Figure 10:**
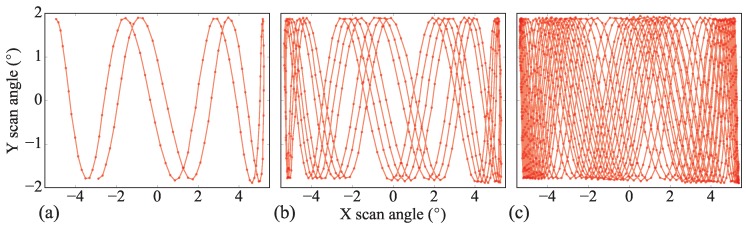
Time elapsed Lissajous laser scanning patterns recorded by the high speed camera at 30 kfps, after (**a**) 4 ms. (**b**) 17 ms. (**c**) 45 ms.

Finally, Lissajous laser scans have been recorded by the high-speed camera (at 30 kfps) when the micromirror is actuated at its resonance frequencies. Corresponding time elapsed scans are shown in Figure 10 (after 4 ms, 17 ms and 45 ms). In these conditions, if, on the one hand, a resolution of 10 μm is sought at a working distance of 5 mm from the mirror as in [4], and on the other hand, a 90 kHz A-scan rate swept-source is employed (requiring to interpolate the 30 kHz experimental scans), it would then require 45 ms, corresponding to an imaging frequency of 22 Hz, to cover 99% of a scanned area of 770 μm × 270 μm. At this frequency, and because of the Lissajous type of scanning, a significant number of pixels is averaged. Larger averaging, e.g., when 95% of the scanned area is illuminated more than 9 times, can be reached at a frequency of 5 Hz.

## Abbreviations

The following abbreviations are used in this manuscript:

MEMS	Micro Electro-Mechanical System
MOEMS	Micro Optical Electro-Mechanical System
SCS	Single Crystal Silicon
OCT	Optical Coherence Tomography
SS	Swept Source
ISC	Inverted Series Connected
MISC	Meshed ISC
SOI	Silicon On Insulator
BOX	Buried Oxide
BOE	Buffered Oxide Etch
PECVD	Plasma-Enhanced Chemical Vapor Deposition
CTE	Coefficient of Thermal Expansion
RIE	Reactive-Ion Etching
ICP	Inductive Coupled Plasma
GRIN	GRadient INdex
PSD	Position Sensing Detector
